# Improving Multidisciplinary Team Documentation and Handover in a Long-Stay Dementia Psychiatry Unit: A Quality Improvement Project

**DOI:** 10.1192/bjo.2026.11435

**Published:** 2026-06-30

**Authors:** Simona Mihalikova, Christina Wong, Kunle Ashaye

**Affiliations:** Hertfordshire Partnership University Trust, Stevenage, United Kingdom

## Abstract

**Aims:**

In long-stay dementia psychiatry settings, multidisciplinary team (MDT) documentation frequently accumulates extensive historical detail, which can obscure current clinical priorities, hinder longitudinal tracking of progress, and reduce clarity around accountability for care planning. Improving the structure and accessibility of MDT records is therefore essential for maintaining continuity of care in complex, chronic inpatient populations. This Quality Improvement Project (QIP) aimed to enhance the clarity, structure, and continuity of weekly MDT documentation in a long-stay old age psychiatry unit. Specific objectives were to ensure clear differentiation of current and resolved issues with explicit progress tracking, consistent use of closed-loop communication with named responsibility and timeframes, and improved accessibility of MDT plans to support ward handover.

**Methods:**

Baseline analysis assessed the presence of core clinical information, identification of current issues, progress tracking, and action accountability within MDT documentation. Astructured MDT template was co-designed with the multidisciplinary team to prioritise current issues, document progress using a standardised framework (better/same/worse), and assign actions to named individuals or teams with review dates. Weekly MDT summary sheets were produced and made available in the nurses’ office to support continuity during daily handovers. Interventions were implemented iteratively using Plan–Do–Study–Act cycles, with re-audit following each cycle.

**Results:**

At baseline, MDT documentation was highly inconsistent, with key clinical elements recorded in only 0–15% of cases. Following introduction of the structured template, completion of core documentation fields increased to 84–100% after the first cycle and reached 100% across all measured domains by the second cycle. Accountability measures improved markedly, with documentation of responsible teams increasing from 1.67% at baseline to 100%, and inclusion of review dates rising from 0% to 100%. The introduction of closed-loop communication and weekly MDT summaries improved the clarity of MDT plans and facilitated information transfer to ward staff, evidenced by unprompted use of summaries by nursing staff during handovers.

**Conclusion:**

This QIP demonstrates that a simple, structured approach to MDT documentation can produce substantial and sustained improvements in clarity, accountability, and continuity of care within a long-stay dementia psychiatry unit. The interventions were low-cost, acceptable to staff, and readily integrated into routine practice. These findings support the wider applicability of structured MDT documentation to improve care coordination in long-term psychiatric inpatient settings, although further work is required to assess sustainability and patient-centred outcomes.

